# Targeting Ras-ERK cascade by bioactive natural products for potential treatment of cancer: an updated overview

**DOI:** 10.1186/s12935-022-02666-z

**Published:** 2022-08-08

**Authors:** Eunus S. Ali, Shamima Akter, Sarker Ramproshad, Banani Mondal, Thoufiqul Alam Riaz, Muhammad Torequl Islam, Ishaq N. Khan, Anca Oana Docea, Daniela Calina, Javad Sharifi-Rad, William C. Cho

**Affiliations:** 1 College of Medicine and Public Health, Flinders University, Bedford Park, 5042 Australia; 2 Department of Bioinformatics and Computational Biology, George Mason University, Fairfax, VA 22030 USA; 3 Department of Pharmacy, Ranada Prasad Shaha University, Narayanganj, 1400 Bangladesh; 4 Department of Pharmacology and Institute of New Drug Development, Jeonbuk National University Medical School, Jeonju, 54907 Republic of Korea; 5 Department of Pharmacy, Life Science Faculty, Bangabandhu Sheikh Mujibur Rahman Science and Technology University, Gopalganj, 8100 Bangladesh; 6 Institute of Basic Medical Sciences, Khyber Medical University, Peshawar, 25100 Pakistan; 7 Department of Toxicology, University of Medicine and Pharmacy of Craiova, 200349 Craiova, Romania; 8 Department of Clinical Pharmacy, University of Medicine and Pharmacy of Craiova, 200349 Craiova, Romania; 9 Facultad de Medicina, Universidad del Azuay, Cuenca, Ecuador; 10 Department of Clinical Oncology, Queen Elizabeth Hospital, Kowloon, Hong Kong

**Keywords:** Cancer, MAPK (ERK), Ras signaling, Ras oncogenes, Molecular mechanisms, Natural bioactive compounds, Chemoresistance, Chemoprevention

## Abstract

MAPK (mitogen-activated protein kinase) or ERK (extracellular-signal-regulated kinase) pathway is an important link in the transition from extracellular signals to intracellular responses. Because of genetic and epigenetic changes, signaling cascades are altered in a variety of diseases, including cancer. Extant studies on the homeostatic and pathologic behavior of MAPK signaling have been conducted; however, much remains to be explored in preclinical and clinical research in terms of regulation and action models. MAPK has implications for cancer therapy response, more specifically in response to experimental MAPK suppression, compensatory mechanisms are activated. The current study investigates MAPK as a very complex cell signaling pathway that plays roles in cancer treatment response, cellular normal conduit maintenance, and compensatory pathway activation. Most MAPK inhibitors, unfortunately, cause resistance by activating compensatory feedback loops in tumor cells and tumor microenvironment components. As a result, innovative combinatorial treatments for cancer management must be applied to limit the likelihood of alternate pathway initiation as a possibility for generating novel therapeutics based on incorporation in translational research. We summarize current knowledge about the implications of ERK (MAPK) in cancer, as well as bioactive products from plants, microbial organisms or marine organisms, as well as the correlation with their chemical structures, which modulate this pathway for the treatment of different types of cancer.

## Introduction

Cancer is the abnormal and anarchic development of cells, with the potential to invade or spread to various parts of the body [1, 2]. Cancer can occur in any part of the body, and cancer cells can invade other organs in different ways (neighbourhood invasion, hematogenous or lymphatic) [3–5]. In 2020, there have been an estimated 18.1 million cancer cases worldwide. Men accounted for 9.3 million of the cases, while women accounted for 8.8 million. Because of recent advances in novel therapeutics, the diagnosis rate has been increasing in recent years, which has improved the general life expectancy for patients [6, 7]. Each cancer type can be subdivided now based on mutations of several genes with the aid of advances in molecular diagnostics [8–10], indicative of the molecular patterns that are malfunctioning. Consequently, this allows effective intervention with tailored treatments by blocking certain biological processes of tumor cells which allows effective interference with targeted therapeutics by impeding specific biological pathways of tumor-infected cells [9, 11]. Cancer is frequently associated with the number of mutations that interrupt the key signaling pathways [12–14]. Cellular signaling pathways are organized as modular networks that communicate in real-time [15, 16]. Pathway components work together in a switch-like fashion, with interactions between two proteins that result in either indirect or direct inhibition or activation of the next factor [17–20]. The pathogenesis of numerous signaling pathways is maintained by transcriptomic, epigenetic, and genetic changes [21, 22].

Nowadays, molecular diagnostic tools became more widely available in clinical settings and that help identifies specific mutational patterns of cancer [23, 24]. Subsequently, this condition becomes an effective method for identifying patients with similar alterations, which was used to determine effective treatment modules [22, 25, 26]. Despite this progress, resistance to cancer therapy remains the main issue i.e., common side effects in patients who have received first-line treatment. Targeted therapy, which employs a variety of small molecules that play a role as inhibitors for the key signaling stages, can result in resistance in a few instances even from first doses. Resistance develops as a consequence of tumor cells being positively designated for mechanisms that can compensate for the specifically targeted pathway [18, 27, 28].

Cancer cells have a low dependence on external proliferative stimuli and often do not need such stimulation to multiply; through the mutations of some oncogenes, these cells acquire a proliferative autonomy, producing their mitogenic signals [29]. One of the key characteristics of cancer cells is probably their ability to permanently stimulate their growth and proliferation [30]z. To understand this property, it is useful to remember that normal cells need the growth and division of external mitogenic signals (mainly represented by growth factors), produced (in a diffusible form) by other cells (paracrine signaling). These molecules (ligands) bind to specific transmembrane receptors, which, after activation, transmit the signal—through branched intracellular signaling pathways—to the nucleus, triggering division. The whole process is regulated by negative feedback mechanisms, which attenuate excessive proliferative signaling (make it transient) and, if it persists, induce cell senescence and apoptosis [31].

Cancer cells have a low dependence on external proliferative stimuli and often do not need such stimulation to multiply; through the mutations of some oncogenes, these cells acquire a proliferative autonomy, producing their mitogenic signals [24, 32]. The major strategies used by tumor cells to achieve proliferative independence are as follows:

- Production of their growth factors, to which they respond by proliferation (autocrine signaling) (for example, TGF-α in sarcomas) [33]
- Disorder of growth factor receptors, which transduce proliferative signals inside the cell; Disorder involves either overexpression of receptors in many cancers (e.g., amplification of the HER2/neu receptor in about 30% of breast cancers or EGFR in non-small cell lung cancer) or alteration of their structure, which results in receptor activation and therefore signaling, without ligand (for example, truncated version of EGF receptor) [34].
- Alteration of the components of cytoplasmic signaling pathways, which produce a flow of mitogenic signaling without their stimulation by receptors; for example, the mitogen-activated protein kinase pathway, which consists of RAS → RAF → MEK → MAPK → ERK → FOS proteins, plays a central role in about 25% of human cancers [35].

In addition to their ability to intensely stimulate their growth and proliferation (by activating oncogenes), cancer cells are insensitive to signals that could stop cell division. In normal tissues, multiple antiproliferative signals act, external or internal, which maintain tissue homeostasis [36]. Exogenous inhibitory signals—either soluble (TGF-β) or embedded in the extracellular matrix and the surface of neighbouring cells (by cadherin-like adhesion molecules, which cause “contact inhibition”) 2—are received by transmembrane receptors coupled with intracytoplasmic circuits. signaling, which blocks cell division. Cells move from the postmitotic G1 stage to the resting G0 phase, from where they can return to the cell cycle—when conditions allow—or permanently give up division and differentiate into specific cells [37].

**Fig. 1:**
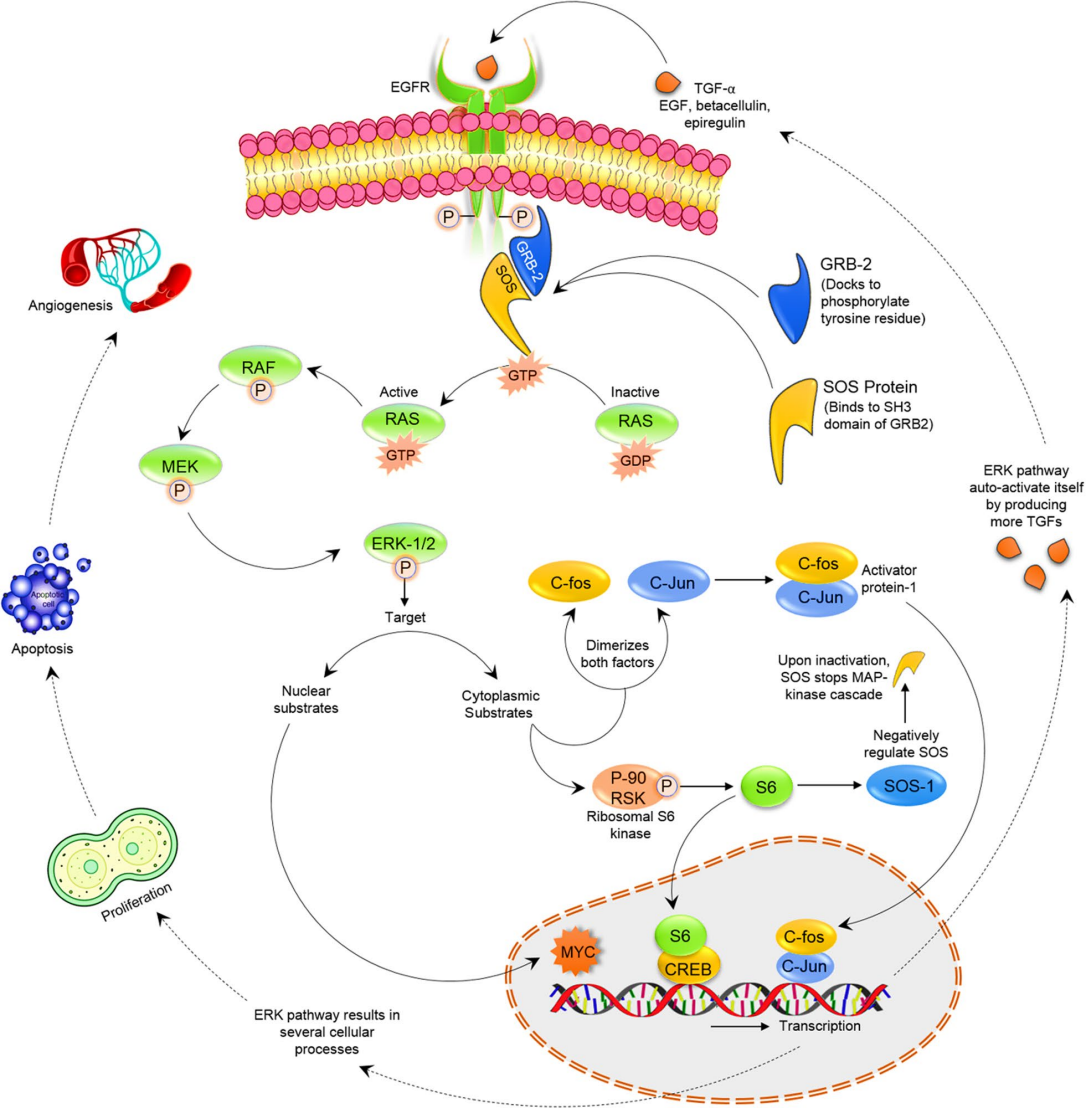
Diagram with involved canonical ERK pathways in primary signal transduction and their associated cellular processes

Complex like MAPK is one compound that linked signaling cascade with frequent participation in tumor development, oncogenesis, and resistance of the drug [38, 39]. The MAPK family includes a large number of kinases that are altered in cancer and for which several targeted therapies have been developed [40]. Resistance to MAPK inhibitors is a current issue, owing to the high degree of interactions and perhaps compensating responses. Thus, in this review, we look at the many repercussions of MAPK pathways in cancer, with a specific emphasis on tumor signaling regulation via MAPK interaction with critical signaling pathways in pathological situations [8]. The current review will concentrate particularly on the pathways of the canonical primary signal transduction depicted in Fig. 1.

## Review methodology

This review has been based on the use of several databases such as PubMed/Medline, Web of Science, TRIP Database, and Up-to-Date using for searching the next MeSH terms: “Antineoplastic Agents/pharmacology”, “Biological Products/chemistry”, “Biological Products/therapeutic use”, “Cell Line”, “MAP Kinase Signaling System/drug effects”, “Extracellular Signal-Regulated MAP Kinases/metabolism”, “Mitogen-Activated Protein Kinase Kinases/antagonists and inhibitors”, “Mitogen-Activated Protein Kinase Kinases/metabolism”, “Neoplasms/drug therapy”, “Neoplasms/metabolism”, **“**Neoplasms/pathology”, “Plants/chemistry”, “Proto-Oncogene Proteins p21(Ras)”, “Signal Transduction/drug effects”, “Ras Proteins/antagonists and inhibitors”, “Ras Proteins/genetics”, “Ras Proteins/metabolism”, “Xenograft Model Antitumor Assays”.

The study included papers published in English that contained molecular pharmacological data on the anticancer action of the phytochemicals mentioned in our study, studies with broad therapeutic perspectives of application, and studies with a high rate of citations. Studies published in languages other than English, studies without obvious pharmacological mechanisms, and studies that included homeoopathic preparations as complementary treatment were excluded.

## Ras-ERK and natural bioactive compounds in chemotherapy and chemoprevention: structure—activity relationship and experimental evidence

Phytochemicals and similar derivative compounds have been shown to play an essential role as cancer treatment agents [2, 41–43]. The majority of protein kinase inhibitors found in plants are flavonoids, which are polyketides. Anthraquinones and anthrones, which contain representatives such as hypericin and emodin, are also part of this chemical group. Both compounds have been shown to inhibit protein kinases like CK2 and p65lck [44]. With alkaloids, most of the flavonoid group are valuable “folk medicine” effective for a variety of ailments other than cancer. However, even when proved to be beneficial in the treatment of cancer, various mechanisms other than intervening with protein kinases have been reported [45, 46].

The extracts of wine polyphenol inhibit the cell cycle progression, cause apoptosis via the caspase activation, and alter the activity of the metalloproteinase (MMP) enzyme. Resveratrol, which is stilbene present in lots of foods including red wine grapes, is thought to provide several health advantages. Protein kinases including MEK/ERK1/2, AKT, RAF, JNK and CamKK, have been demonstrated to be inhibited by it. As a result, it is tempting to infer that the reported “anticancer effects” are due to interfering with the different protein kinases. However, considering the modest levels of resveratrol absorbed by wine intake, this may be more apparent than genuine. Even consuming the ultra-pure resveratrol component should not be enough to have a discernible influence on the activity of cellular protein kinase. Furthermore, considering the relatively high number of distinct protein kinases that are affected by resveratrol, it is impossible to rule out unfavourable side effects when significant doses are used.

A similar argument may be made for the other natural chemicals discussed in this review. An example: secondary metabolites include alkaloids, which are mostly constituted of nitrogen and are commonly employed in medicine [47]. Alkaloids are one of the most diverse classes of natural chemicals, with over 12,000 known structures. The ability of the secondary plant metabolites to inhibit the expression of both non-coding and coding genes is then used to modulate a variety of cellular pathways, including MAPK [48, 49]; Table 1 contains several examples.

**Table 1:** Some examples of naturally occurring biologically active compounds to regulate the MAPK in parallel with another associated pathway involved in cancer invasion and progression

Natural compounds	Type of cancer	Preclinical Model In vitro/cancer cell lines	Molecular targets	Effects	Refs.
Caffeic acid phenethyl ester (CAPE) + U0126	Pancreatic ductal adenocarcinoma	PANC-1 MIAPaCa-2	↓ NF-κB ↓ MAPK	↓ Cell growth, ↑ Apoptosis (PANC-1 caspase-independent mode and MIAPaCa-2 caspase-dependent)	[51]
Apigenin	Choriocarcinoma	JEG3 JAR	↓ ERK1/2 ↓ PI3K/AKT	↓ Migratory capacity ↓ Cell viability, ↑apoptosis	[53]
Coumestrol	Prostate cancer	LNCaP PC3	↓ Phosphorylation of AKT proteins ↑ Phosphorylation f P90RSK, JNK, ERK1/2, p53	↓Cell proliferation ↓migration ↑apoptosis	[54]
Quercetin	Choriocarcinoma	JEG3 JAR	↑ Phosphorylation of p38, JNK, ERK1/2, and P90RSK proteins ↓ Phosphorylation of P70S6K, AKT, S6	↓ Proliferation ↓ Invasion ↓ Cell-cycle progression	[55]
Kaempferol	Endometrial Malignant transformation	EBM-2 HUVECs	↓ VEGFR2 ↓ HIF-1α proteins ↓ Phosphorylation of and p38, ↓ ERK, ↓ Akt	↓ Angiogenesis	[56]
Genistein	Melanoma	B16F10	↓ ERK, ↓ p38, ↓ JNK, ↓ Phosphorylation of tensin-2, ↓ FAK, ↓ paxillin, ↓ vinculin	↓ Cells growth ↓ Cells migration	[57]
Genistein and Novasoy	Endometrial cancer	RL-95–2 ECC-1 cells	↑ Phosphorylation of S6 only in RL-95–2 cells ↑ Phosphorylation of the p42/44 in both cell line	↓ Cellular proliferation ↓ Cell-cycle arrest in G2 phase ↑ Apoptosis	[58]
Resveratrol	T-cell acute lymphoblastic leukemia	Jurkat (glucocorticoid resistant) and T-ALL cell lines, Molt-4 (glucocorticoid resistant)	↑ p38-MAPK ↓ Akt/p70S6K/mTOR/4E-BP1	↑ Autophagy ↑ Apoptosis	[59, 60]
Escine	Osteosarcoma	MNNG, MG-63, Saos-2, U-2OS	↑ p38	↑ Autophagy ↑ Apoptosis	[61]
Triterpenoids (21α-methylmelianodiol)	Lung cancer	A549	↓ ERK, ↓p-JNK, ↓p-ERK, ↓p38, ↓JNK, no effect on p-p38	Targeting drug resistance via P-glycoprotein (P-gp)/MDR1-association	[62]
Toosendanin	Lung cancer	H1975 and A549 cells	↓ Snail, ↓TGFβ1, ↓ Phosphorylation of ERK	Prevents TGFβ1-induced EMT and invasion, migration, and adhesion	[63]
Luteolin	Cervical cancer	Hela cells	↑ Fas, ↑ phospho-JNK, ↑ p53, ↑ phospho-p38, ↑ Bax, ↓ PARP, ↓ mTOR, ↓ Bcl-2	↓ Cellular proliferation ↑ Apoptosis	[64]
Baicalein	Hepatocellular carcinoma	HepG2cell xenograft in nude mice	↓ MEK1 ↓ Bad ↓ ERK1/2	↑ Intrinsic apoptosis	[65]
Fisetin	Laryngeal cancer	TU212 cell	↓ RAS ↓ RAF ↓ ERK1/2	↓ Cell migration ↓ Proliferation	[66]
Naringenin	Prostate cancer	LNCaP and PC3 cells	↓ p38 ERK1/2, ↓ S6, ↓ P70S6K, ↓ JNK	↑ Apoptosis, ↑ ROS ↓ Proliferation ↓ Migration	[67]
Silibinin	Hepatocellular carcinoma	Bel-7404 xenografts in nude mice Bel-7404	Combined treatment with the sorafenib ↓ Phosphorylation of ERK, STAT3, AKT, MAPK p38	↓ Proliferation ↑ Apoptosis	[68, 69]
Taxifolin	Skin cancer	skin carcinogenesis mouse model, JB6 Pþ mouse skin epidermal cells	↓ Phosphorylation of p38, EGFR, ERKs, JNKs	↓ Tumor incidence, ↓ Multiplicity in a solar UV (SUV)-induced skin carcinogenesis	[70]
Delphinidin	Osteosarcoma	HOS, U2OS, MG-63 cells	↓ Phosphorylated forms of p38 ↓ ERK	↓ Cell migration ↓ EMT ↓ Cellular proliferation ↑ Apoptosis	[71]
Parthenolide	Non-small cell lung cancer	GLC-82 cells	↓ c-Myc, ↓ B-Raf, ↓ Phosphorylation of Erk, MEK,	↓ Invasion ↓ Proliferation ↑ Apoptosis	[72, 73]
Oridonin	Esophageal cancer	KYSE-150 c xenograft KYSE-150 cancer nude mice	↓ Ras/Raf/MEK/ERK ↓ EGFR-mediated PI3K/AKT	↓ Tumor angiogenesis ↓ Angiogenesis-marker CD31 ↑ Apoptosis	[74]
Curcumin	Lung and pancreatic adenocarcinoma	p34, H1299, PC-14, Panc1	↓ Erk1/2 ↓ COX-2, ↓ EGFR	↓ Survival of cancer cell ↑ Apoptosis	[75]
Licochalcone A	Human gastric cancer	BGC-823	↑ JNK, ↑ ERK, ↑ p38 MAPK	↑ Oxidative stress ↑ Apoptosis	[76]
Pterostilbene	Breast cancer	MCF-7 MDA-MB-231	↓ Akt, ↓ ERK1/2	↑ Apoptosis ↓ Proliferation	[77]
Arctigenin	Gallbladder cancer	GBC-SD, NOZ GBC-SD	↓ EGFR, ↓ p-b-Raf, ↓ p–c-Rafp-MEK, ↓ ERK, ↓ MEK, ↓ p-AKT, ↓AKT	↑ Cancer senescence	[78]
α-mangostin	Cervical cancer	SiHa and HeLa cells and xenograft model	↑ p-ASK1, p-p38 p-MKK3/6	↑ Apoptosis	[79]
Vitisin A	Pro-tumorigenic inflammation	RAW 264.7 cells	↓ p38, ↓ERK, ↓ NF-κB	↓ Proliferation	[80]
Azaspirene	Renal carcinoma	Renal carcinoma xenograft model HUVEC	↓ Raf‐1	↓ Angiogenesis	[81]
Rocaglamide	Leukemia	Jurkat leukemic cells	↓ Raf-MEK-ERK	Targeting prohibitin 1 and 2	[82]
L-783277	Human pancreatic cancer	PSN1	↓ Phosphorylation of Ras-dependent MAP kinase	↓ Proliferation	[83]
Magnolin	Non-small cell lung carcinoma	NCI-H1975 A549	↓ ERKs/RSK2	↓NF-κB	[84]
Tomatidine	Sarcoma	HT1080	↓ ERK ↓ p38	↓ p38, ↓ ERK ↓ Modulation of gelatinase	[85]
Catechol	Lung cancer	H460 KP2	↓ ERK2	↑ c-Myc degradation ↓ ERK2	[86]
1,2,3-Triazole Curcumin	Non-small cell lung carcinoma	A549	↓ NF-κB/STAT3 ↑ mitogen-activated protein kinases	↓ Cell proliferation	[87]

↑ increase, ↓ decrease, *ROS* reactive oxygen species; *T-ALL* T-cell acute lymphoblastic leukemia; *HIF* Hypoxia-inducible factors; *JNKs* c-Jun N-terminal kinases; *TGFβ* transforming growth factor-beta; *ERK* extracellular regulated MAP kinase; *p38* p38 kinase; *AKT* v-akt murine thymoma viral oncogene homolog 1; *VEGFR* vascular endothelial growth factor

For the suppression of cell proliferation in pancreatic cells, the NF-kB and MAPK survival pathways were empirically suppressed using CAPE (caffeic acid phenethyl ester) and U0126. CAPE only activated the apoptosis mechanism after autophagy was inhibited [48, 50]; in MIAPaCa-2 cells, this occurred in a caspase-dependent fashion, but in PANC-1 cells, it occurred in a caspase-independent mode [51]. CAPE is a complicated therapeutic agent that affects not only programmed cell death but also angiogenesis and EMT pathways [52]. Furthermore, this study emphasizes the significance of selecting the appropriate cell culture model and understanding the characteristics of the cell lines to collect meaningful results [51].

Apigenin, a flavonoid is another chemopreventive drug that suppresses the development of choriocarcinoma cells by regulating the ERK1/2 MAPK and PI3K/AKT signal transduction mechanisms. The presence of ERK1/2 inhibitors and PI3K/AKT inhibitors enhances these effects [53].

Kaempferol, another flavanol, has been linked to angiogenesis inhibition by affecting HIF-1 and VEGFR2 in endothelial cells via a process involving PI3K/AKT/mTOR and ERK/p38. Endothelial cells were treated with kaempferol in combination with a p38 inhibitor (SB203580) or an ERK inhibitor (PD98059), and the therapeutic effectiveness of kaempferol was found to be enhanced [88].

Coumestrol, phaseol, and isotrifoliol have been shown significant anti-inflammatory effects on LPS (Lipopolysaccharide)-induced RAW264.7 macrophages, mostly via TLR/MAPK signaling and TLR (Toll-like receptors)/NF-kB [89]. Coumestrol, a phytoestrogen, inhibits cell proliferation through modulating MAPK-related genes and an AKT-related compensatory mechanism [54]. Quercetin, a flavonol molecule found in high concentrations, has been shown to inhibit choriocarcinoma growth by interfering with PI3K and MAPK signal transduction. Furthermore, quercetin enhanced the chemotherapeutic effects of paclitaxel and cisplatin in the cell lines of choriocarcinoma (JEG3 and JAR) [55].

Isoflavones were recently thought to be promising anti-cancer medicines [90]. The consequences of genistein and novasoy were studied in endometrial cancer cells. These cells were found to have an antiproliferative impact associated with the activation of the MAPK and AKT/mTOR signaling pathways [58]. Furthermore, it has been established that genistein can reduce ER expression while increasing PR (progesterone receptor) expression [58]. In melanoma cells, genistein inhibits the proliferation of cells, migration and invasion, via the MAPK and FAK/paxillin pathways [57]. Furthermore, 5,6,7,3′,4′,5′-hexamethoxyflavone which is a polymethoxyflavone has been demonstrated to impede the cellular proliferation of triple-negative breast cancer (through MAPK/AKT targeting) and cell-cycle arresting [91].

Resveratrol is a controversial natural chemical with anti-tumour properties that is being investigated as a possible treatment possibility [92]. Resveratrol can preferentially trigger autophagy and apoptosis in the T-cell acute lymphoblastic leukaemia cells via suppression of mTOR/AKT/4E-BP1/p70S6K and initiation of p38-MAPK pathways [93].

Escin, a combination of triterpene and saponins derived from the *Aesculus hippocastanum*, has anti-tumor ability via autophagy and apoptosis regulation via ROS/p38 MAPK signaling [94].

Furthermore, 21-methylmelianodiol (21-MMD) obtained from *Poncirus trifoliata*, has anti-tumor effect in the cancer of the lung via interfering with MAPK signaling and AKT/ PI3K/AMPK signaling it is also related to multi-drug resistance reversal by reducing the expressions of P-gp/MDR1 (P-glycoprotein/multidrug resistance protein 1) [95].

Toosendanin is a natural insecticide that has been shown in lung cancer models to switch EMT markers expression via ERK/Snail signaling pathway [96].

Sulforaphane (SFN) is another natural chemical that has been studied for its potential use in the treatment of OS (osteosarcoma) [97]. This isothiocyanate chemical is derived from vegetables such as broccoli, Brussels sprouts, and cabbage. Sawai et al. examined the effects of SFN on the cell line of *murine* osteosarcoma (LM8)*.* These cells were grown with SFN at various doses, which caused improved cell populations in the phase of G2/M. The combination of 2 Gy of radiation and SFN inhibited the phosphorylation of ERK and AKT. SFN was also shown to cause apoptosis via G2/M phase arrest and to decrease ERK and AKT activation [98]. Another study found that SFN caused genomic instability in the cell lines of MG63 OS by mitotic and nuclear abnormalities, clastogenicity and DNA breaks. Increased production of micronuclei and apoptotic bodies indicated viability loss. SFN might be an effective molecular targeting chemotherapeutic drug for ovarian cancer [99].

**Fig. 2:**
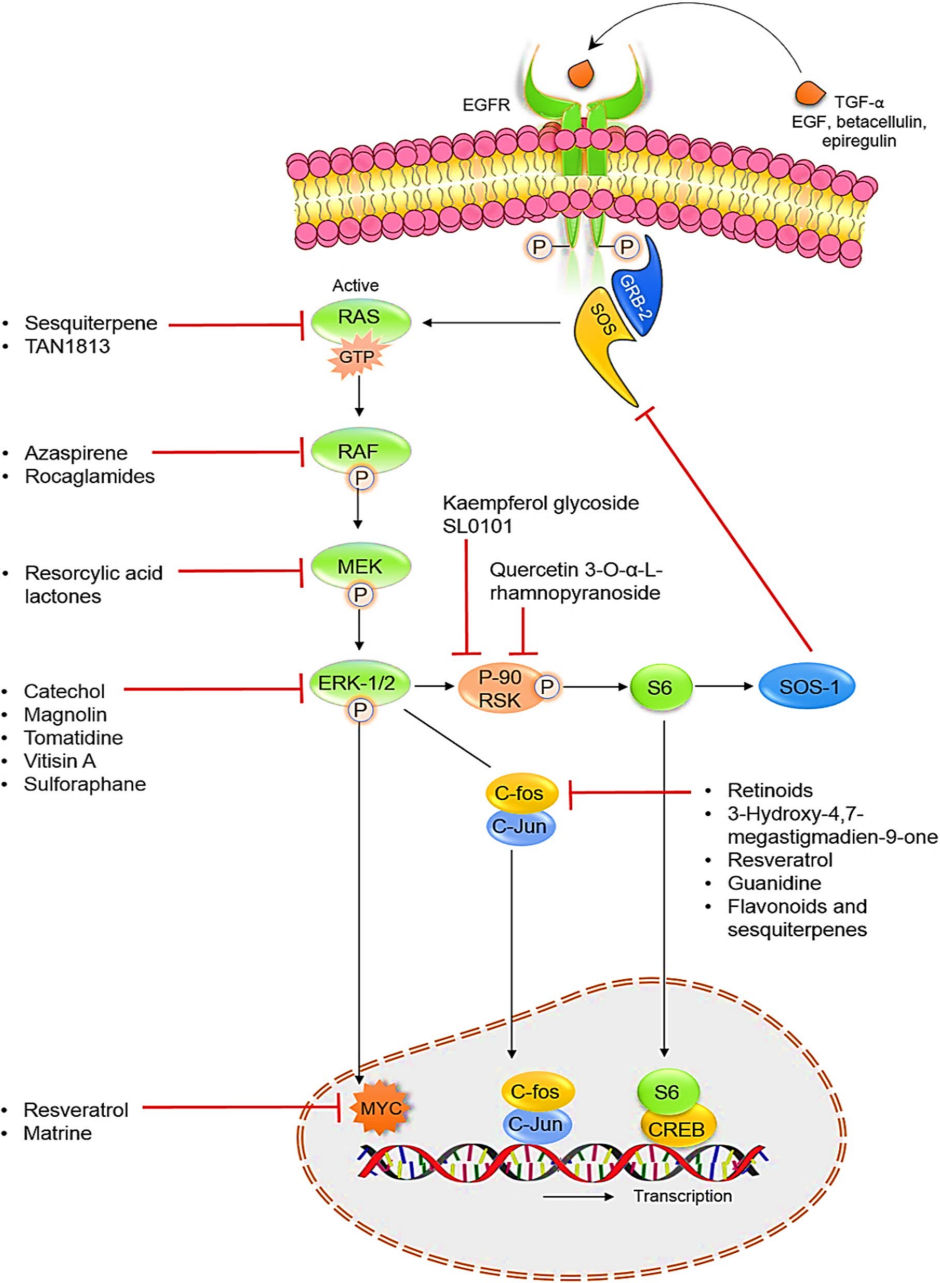
Depiction of some of the bioactive natural products and their important areas of action in the Ras-ERK cascade

Figure 2 depicts some of the most essential areas of action of those natural chemicals and Table 1 presents the most representative chemical structures of bioactive compounds which modulate the Ras-ERK cascade.

## Natural bioactive compounds as kinase inhibitors

Plants have a vast store of natural bioactive compounds with beneficial effects on human health [46, 100–102]. According to one study, 80% of the world's population still uses plant-derived medications to meet their healthcare needs [45, 103, 104]. Traditional medicine utilizes a combination of many ingredients; however, the ingredients might not show activities as a single entity but sometimes a combination of ingredients plays an important role in having synergistic effects and modulating other proteins which improve the efficacy of the bioactive principle [105–107]. Protein kinase inhibitors have recently been shown to be chemically linked to a class of plant chemicals known as sesquiterpenes, alkaloids, flavonoids, polyphenolics, and diterpenoids, which are present in a variety of fruits, vegetables, and medicinal plants and have anti-cancer properties [44, 108]. The FDA authorized 1453 new chemical entities in 2013 with natural products or analogues of natural substances accounting for 40% of the total [109].

DLW (Danggui Longhui Wan) is a common traditional Chinese herbal medicine to treat chronic myeloid leukemia (CML) [110]. It’s made up of eleven different plant ingredients. Only indirubin was shown to be effective against CML during the hunt for the active chemical. The other ten compounds were all inactive. Indirubin is an effective inhibitor of CDKs (cyclin-dependent kinases), which are important in cell division. Because tumor cells rely largely on cell division, inhibiting CDKs will prevent cell division progression and therefore tumor development. Indirubin, on the other hand, performed well but was hard to absorb in the digestive system [111]. Gliotoxin is a sulfur-containing mycotoxin generated by a pathogenic fungus such as *Aspergillus fumigatus* that inhibits Ras protein and hence cell development [112]. Magnolin, a natural chemical present in Magnolia flos, inhibits cell proliferation caused by tumor promoters such as EGF (epidermal growth factor) and focuses on ERK1 and ERK2 [113].

## Discussions

There is proof that the usage of natural compounds originating from microbes, animals, or plants for medical purposes goes back to the Neanderthal epoch [114, 115]. People have gathered knowledge and become an expert in their applications due to various biological activities of isolated natural items [116–118]. The invention of the chemical structure aided the manufacturing of the essential compounds rather than separating them from natural sources [119]. This process was also less expensive and allowed for the use of the active element of the medicinal plant rather than the basic plant extraction [106, 107]. Marketed medications like camptothecin, artemisinin, maytansine, lovastatin, penicillin, paclitaxel, silibinin and reserpine were either indirectly or directly developed from natural compounds [120]. Natural goods are now being viewed as a viable substitute for manufactured medications. These natural compounds can be found in a variety of sources, including plants, microbes, and fungus [118]. Today, pharmaceutical research is shifting away toward multiple target approaches than single-molecule target techniques.

Natural products have been shown to initiate apoptosis and chemosensitive cell lines that were previously resistant to conventional treatments [121–123]. The heterogeneity of natural compounds' inefficiency found in the cell line-centred tests and rodent models used throughout the phase of the drug discovery, which leads to the ultimate effectiveness in patients, is a significant barrier in the creation of a particular inhibitor [124].

Since the first kinase inhibitor was developed in the 1980s, more than 40 kinase inhibitors have been approved by the FDA for the treatment of malignant cells such as lung and breast cancer cells. Furthermore, around 150 kinase-targeted medicines are in clinical trials, and preclinical research is also going on numerous kinase-specific inhibitors [125]. Despite the promising anti-tumour activity and survival improvements gained by licensed RAF, MEK, and ERK inhibitors, drug resistance is the main limitation of the development of new MAPK pathway inhibitors [125]. The underlying processes, which are often associated with genomic instability and cancer heterogeneity, are largely associated with the compensatory initiation of the upstream component. More research into the MAPK pathway has led to the hypothesis that targets the downstream kinase of the ERK, and also the combination of ERK inhibition with MEK and RAF inhibition may be advantageous [125].

B-Raf is in therapeutic use among a few serine/threonine kinases inhibitors. These inhibitors of serine/threonine kinase for MAPK Aurora kinases, and CK2 and mTOR are being developed for therapeutic use [126]. We concentrated on the pathways of the canonical primary signal transduction depicted in Fig. 1. The emphasis is primarily on natural products made from plant sources, particularly flavonoids. Protein kinase B, cyclin-dependent kinases, polo-like kinase I, and other enzymes are affected. CDKs have also been targeted using natural chemicals derived from sources other than plants, such as marine creatures. New microorganisms that can survive in extreme environmental conditions called “extremophiles” have opened new perspectives in the biotechnology/pharmaceutical industry with anti-cancer therapeutic potential by blocking the cell cycle. They also have antioxidant effects. It has been observed that most extremophilic microorganisms have an increased resistance to ultraviolet radiation and can be used to develop anticancer drugs [127]. This might be a new source for developing more strong kinase inhibitors.

## Limitations and future perspectives

This review has some limitations that need to be addressed in the future, such as the lack of in vivo studies that provide mechanistic perspectives on molecular interactions and targets of action of phytochemicals included in this study. Also, future in silico studies such as molecular docking may address molecular targets for a better understanding of phytochemical interactions in different signaling pathways.

Another limitation is the lack of large-scale and well-controlled clinical trials to validate the efficacy of these molecular targets, their adverse effects, and the safety of their administration for the treatment of cancer.

Although natural products show excellent results in vitro, the development of cytostatic drugs in them is a complex process, except for a few. The therapeutic advantage of these natural products is their minimal toxicity and reduced side effects [92, 128, 129]. But natural bioactive compounds can interact with many proteins, which is why it is very important to elucidate the mechanisms of action of natural products, especially those that are used in our diet [130].

In recent decades, kinase inhibitors have received more attention in search of new drugs and bioactive natural compounds that target a wide range of kinases. However, there are some therapeutic limitations of natural bioactive compounds, such as poor solubility, complexity, and biodisponibility [131–133]. Therefore, to overcome these clinical pitfalls and to obtain food supplements officially approved by the competent authorities, a comprehensive quality analysis must be performed in terms of bioavailability, efficacy, safety, composition, technological manufacturing processes, pharmaceutical regulatory practices and compliance with international standards.

## Conclusion

At the time, too many diverse biological features are known, making it easy to infer that they are to blame for the reported health impacts. Each ingredient in plant extracts is not effective enough to account for successful cancer treatment on its own. There is a growing body of evidence that protein kinase inhibitors can be isolated from sources other than plants. Protein kinase inhibitors have recently been obtained from marine sources. This is a novel and promising strategy for discovering new forms of kinase inhibitors. However, there are a lot of other factors, such as genetics, environment, physical activity, dietary habits, and so on, and food-related considerations alone are insufficient. When a specific molecule would be targeted, which may pave the door for larger use of flavonoids, simply because they interfere with multiple cellular ‘war fields’. When employed appropriately, this finding might lead to an effective anticancer treatment in the future.

## Data Availability

Yes.

## References

[1] Sell S, Nicolini A, Ferrari P, Biava PM (2016). Cancer: a problem of developmental biology; scientific evidence for reprogramming and differentiation therapy. Curr Drug Targets.

[2] Sharifi-Rad J, Quispe C, Bouyahya A, El Menyiy N, El Omari N, Shahinozzaman M, Ara Haque Ovey M, Koirala N, Panthi M, Ertani A (2022). Ethnobotany, phytochemistry, biological activities, and health-promoting effects of the Genus Bulbophyllum. Evid Based Complement Alternat Med..

[3] Quetglas-Llabrés MM, Quispe C, Herrera-Bravo J, Catarino MD, Pereira OR, Cardoso SM, Dua K, Chellappan DK, Pabreja K, Satija S (2022). Pharmacological properties of bergapten: mechanistic and therapeutic aspects. Oxid Med Cell Longev.

[4] GBD 2019 Colorectal Cancer Collaborators (2022). Global, regional, and national burden of colorectal cancer and its risk factors, 1990–2019: a systematic analysis for the Global Burden of Disease Study 2019. Lancet Gastroenterol Hepatol.

[5] Kato Y, Maeda T, Suzuki A, Baba Y (2018). Cancer metabolism: new insights into classic characteristics. Jpn Dent Sci Rev.

[6] Sharifi-Rad J, Quispe C, Patra JK, Singh YD, Panda MK, Das G, Adetunji CO, Michael OS, Sytar O, Polito L (2021). Paclitaxel: application in modern oncology and nanomedicine-based cancer therapy. Oxid Med Cell Longev.

[7] Docea AO, Mitrut P, Grigore D, Pirici D, Calina DC, Gofita E (2012). Immunohistochemical expression of TGF beta (TGF-beta), TGF beta receptor 1 (TGFBR1), and Ki67 in intestinal variant of gastric adenocarcinomas. Rom J Morphol Embryol.

[8] Braicu C, Buse M, Busuioc C, Drula R, Gulei D, Raduly L, Rusu A, Irimie A, Atanasov AG, Slaby O (2019). A comprehensive review on MAPK: a promising therapeutic target in cancer. Cancers.

[9] Cainap C, Nagy V, Seicean A, Gherman A, Laszlo I, Lisencu C, Nadim AH, Constantin A-M, Cainap S (2016). Results of third-generation epirubicin/cisplatin/xeloda adjuvant chemotherapy in patients with radically resected gastric cancer. J BUON.

[10] Jain D, Chaudhary P, Varshney N, Bin Razzak KS, Verma D, Zahra TRK, Janmeda P, Sharifi-Rad J, Dastan SD, Mahmud S (2021). Tobacco smoking and liver cancer risk: potential avenues for carcinogenesis. J Oncol.

[11] Mitrut P, Docea AO, Kamal AM, Mitrut R, Calina D, Gofita E, Padureanu V, Gruia C, Streba L (2016). Colorectal cancer and inflammatory bowel disease.

[12] Dhyani P, Quispe C, Sharma E, Bahukhandi A, Sati P, Attri DC, Szopa A, Sharifi-Rad J, Docea AO, Mardare I (2022). Anticancer potential of alkaloids: a key emphasis to colchicine, vinblastine, vincristine, vindesine, vinorelbine and vincamine. Cancer Cell Int.

[13] Semwal P, Painuli S, Abu-Izneid T, Rauf A, Sharma A, Daştan SD, Kumar M, Alshehri MM, Taheri Y, Das R (2022). Diosgenin: an updated pharmacological review and therapeutic perspectives. Oxid Med Cell Longev.

[14] Zijlstra A, Von Lersner A, Yu D, Borrello L, Oudin M, Kang Y, Sahai E, Fingleton B, Stein U, Cox TR (2019). The importance of developing therapies targeting the biological spectrum of metastatic disease. Clin Exp Metastasis.

[15] Buga AM, Docea AO, Albu C, Malin RD, Branisteanu DE, Ianosi G, Ianosi SL, Iordache A, Calina D (2019). Molecular and cellular stratagem of brain metastases associated with melanoma. Oncol Lett.

[16] Kato Y, Ozawa S, Miyamoto C, Maehata Y, Suzuki A, Maeda T, Baba Y (2013). Acidic extracellular microenvironment and cancer. Cancer Cell Int.

[17] Plotnikov A, Zehorai E, Procaccia S (1813). Seger R (2011) The MAPK cascades signaling components, nuclear roles and mechanisms of nuclear translocation. Biochim Biophys Acta Mol Cell Res.

[18] Liu F, Yang X, Geng M, Huang M (2018). Targeting ERK, an Achilles' heel of the MAPK pathway, in cancer therapy. Acta Pharm Sin B.

[19] Ali ES, Rychkov GY, Barritt GJ (2020). Targeting Ca(2+) signaling in the initiation, promotion and progression of hepatocellular carcinoma. Cancers (Basel).

[20] Sutoo S, Maeda T, Suzuki A, Kato Y (2020). Adaptation to chronic acidic extracellular pH elicits a sustained increase in lung cancer cell invasion and metastasis. Clin Exp Metastasis.

[21] Sanchez-Vega F, Mina M, Armenia J, Chatila WK, Luna A, La KC, Dimitriadoy S, Liu DL, Kantheti HS, Saghafinia S (2018). Oncogenic signaling pathways in the cancer genome atlas. Cell.

[22] Seles M, Hutterer GC, Kiesslich T, Pummer K, Berindan-Neagoe I, Perakis S, Schwarzenbacher D, Stotz M, Gerger A, Pichler M (2016). Current insights into long non-coding RNAs in renal cell carcinoma. Int J Mol Sci.

[23] Zlatian OM, Comanescu MV, Rosu AF, Rosu L, Cruce M, Gaman AE, Calina CD, Sfredel V (2015). Histochemical and immunohistochemical evidence of tumor heterogeneity in colorectal cancer. Rom J Morphol Embryol.

[24] Ianoși SL, Batani A, Ilie MA, Tampa M, Georgescu SR, Zurac S, Boda D, Ianosi NG, Neagoe D, Calina D (2019). Non-invasive imaging techniques for the in vivo diagnosis of Bowen's disease: three case reports. Oncol Lett.

[25] Braicu C, Catana C, Calin A G, Berindan-Neagoe I (2014). NCRNA combined therapy as future treatment option for cancer. Curr Pharm Des.

[26] Braicu C, Zimta A-A, Harangus A, Iurca I, Irimie A, Coza O, Berindan-Neagoe I (2019). The function of non-coding RNAs in lung cancer tumorigenesis. Cancers.

[27] Braicu C, Pileczki V, Irimie A, Berindan-Neagoe I (2013). p53siRNA therapy reduces cell proliferation, migration and induces apoptosis in triple negative breast cancer cells. Mol Cell Biochem.

[28] Ganapathi MK, Jones WD, Sehouli J, Michener CM, Braicu IE, Norris EJ, Biscotti CV, Vaziri SA, Ganapathi RN (2016). Expression profile of COL2A1 and the pseudogene SLC6A10P predicts tumor recurrence in high-grade serous ovarian cancer. Int J Cancer.

[29] Fouad YA, Aanei C (2017). Revisiting the hallmarks of cancer. Am J Cancer Res.

[30] Sani TA, Mohammadpour E, Mohammadi A, Memariani T, Yazdi MV, Rezaee R, Calina D, Docea AO, Goumenou M, Etemad L (2017). Cytotoxic And apoptogenic properties Of *Dracocephalum*
*Kotschyi* aerial part different fractions on calu-6 and mehr-80 lung cancer cell lines. Farmacia.

[31] Sever R, Brugge JS (2015). Signal transduction in cancer. Cold Spring Harb Perspect Med.

[32] Shortt J, Johnstone RW (2012). Oncogenes in cell survival and cell death. Cold Spring Harb Perspect Biol.

[33] Ungefroren H (2021). Autocrine TGF-β in cancer: review of the literature and caveats in experimental analysis. Int J Mol Sci.

[34] Wee P, Wang Z (2017). Epidermal growth factor receptor cell proliferation signaling pathways. Cancers (Basel).

[35] Pylayeva-Gupta Y, Grabocka E, Bar-Sagi D (2011). RAS oncogenes: weaving a tumorigenic web. Nat Rev Cancer.

[36] Debela DT, Muzazu SG, Heraro KD, Ndalama MT, Mesele BW, Haile DC, Kitui SK, Manyazewal T (2021). New approaches and procedures for cancer treatment: current perspectives. SAGE Open Med.

[37] Silk JD, Abbott RJM, Adams KJ, Bennett AD, Brett S, Cornforth TV, Crossland KL, Figueroa DJ, Jing J, O'Connor C (2022). Engineering cancer antigen-specific T cells to overcome the immunosuppressive effects of TGF-β. J Immunol.

[38] Ali ES, Sahu U, Villa E, O’Hara BP, Gao P, Beaudet C, Wood AW, Asara JM, Ben-Sahra I (2020). ERK2 Phosphorylates PFAS to mediate posttranslational control of de novo purine synthesis. Mol Cell.

[39] Lee S, Rauch J, Kolch W (2020). Targeting MAPK signaling in cancer: mechanisms of drug resistance and sensitivity. Int J Mol Sci.

[40] Cargnello M, Roux PP (2011). Activation and function of the MAPKs and their substrates, the MAPK-activated protein kinases. Microbiol Mol Biol Rev.

[41] Sharifi-Rad J, Quispe C, Butnariu M, Rotariu LS, Sytar O, Sestito S, Rapposelli S, Akram M, Iqbal M, Krishna A (2021). Chitosan nanoparticles as a promising tool in nanomedicine with particular emphasis on oncological treatment. Cancer Cell Int.

[42] Taheri Y, Quispe C, Herrera-Bravo J, Sharifi-Rad J, Ezzat SM, Merghany RM, Shaheen S, Azmi L, Prakash Mishra A, Sener B (2022). Urtica dioica-derived phytochemicals for pharmacological and therapeutic applications. Evid Based Complement Alternat Med.

[43] Painuli S, Quispe C, Herrera-Bravo J, Semwal P, Martorell M, Almarhoon ZM, Seilkhan A, Ydyrys A, Rad JS, Alshehri MM (2022). Nutraceutical profiling, bioactive composition, and biological applications of *Lepidium*
*sativum* L.. Oxid Med Cell Longev.

[44] Baier A, Szyszka R (2020). Compounds from natural sources as protein kinase inhibitors. Biomolecules.

[45] Rajib H, Muhammad Torequl I, Pranta R, Divya J, Abu Saim Mohammad S, Lutfun N, Anupam Das T, Satyajit S, Seyed Abdulmajid A, Miquel M (2021). Amentoflavone, new hope against SARS-CoV-2: an outlook through its scientific records and an in silico study. Pharmacogn Res.

[46] Hossain R, Sarkar C, Hassan SMH, Khan RA, Arman M, Ray P, Islam MT, Daştan SD, Sharifi-Rad J, Almarhoon ZM (2021). In silico screening of natural products as potential inhibitors of SARS-CoV-2 using molecular docking simulation. Chin J Integr Med.

[47] Hossain R, Quispe C, Saikat ASM, Jain D, Habib A, Janmeda P, Islam MT, Radha SD, Kumar M (2022). Biosynthesis of secondary metabolites based on the regulation of microRNAs. Biomed Res Int.

[48] Budisan L, Gulei D, Zanoaga OM, Irimie AI, Sergiu C, Braicu C, Gherman CD, Berindan-Neagoe I (2017). Dietary intervention by phytochemicals and their role in modulating coding and non-coding genes in cancer. Int J Mol Sci.

[49] Braicu C, Mehterov N, Vladimirov B, Sarafian V, Nabavi SM, Atanasov AG, Berindan-Neagoe I (2017). Nutrigenomics in cancer: revisiting the effects of natural compounds. Semin Cancer Biol.

[50] Budisan L, Gulei D, Jurj A, Braicu C, Zanoaga O, Cojocneanu R, Pop L, Raduly L, Barbat A, Moldovan A (2019). Inhibitory effect of CAPE and Kaempferol in colon cancer cell lines-possible implications in new therapeutic strategies. Int J Mol Sci.

[51] Papademetrio DL, Lompardia SL, Simunovich T, Costantino S, Mihalez CY, Cavaliere V, Alvarez E (2016). Inhibition of survival pathways MAPK and NF-kB triggers apoptosis in pancreatic ductal adenocarcinoma cells via suppression of autophagy. Target Oncol.

[52] Gherman C, Braicu OL, Zanoaga O, Jurj A, Pileczki V, Maralani M, Drigla F, Braicu C, Budisan L, Achimas-Cadariu P (2016). Caffeic acid phenethyl ester activates pro-apoptotic and epithelial-mesenchymal transition-related genes in ovarian cancer cells A2780 and A2780cis. Mol Cell Biochem.

[53] Lim W, Park S, Bazer FW, Song G (2016). Apigenin reduces survival of choriocarcinoma cells by inducing apoptosis via the PI3K/AKT and ERK1/2 MAPK pathways. J Cell Physiol.

[54] Lim W, Jeong M, Bazer FW, Song G (2017). coumestrol inhibits proliferation and migration of prostate cancer cells by regulating AKT, ERK1/2, and JNK MAPK cell signaling cascades. J Cell Physiol.

[55] Lim W, Yang C, Park S, Bazer FW, Song G (2017). Inhibitory effects of quercetin on progression of human choriocarcinoma cells are mediated through PI3K/AKT and MAPK signal transduction cascades. J Cell Physiol.

[56] Kim GD (2017). Kaempferol inhibits angiogenesis by suppressing HIF-1alpha and VEGFR2 activation via ERK/p38 MAPK and PI3K/Akt/mTOR signaling pathways in endothelial cells. Prev Nutr Food Sci.

[57] Cui S, Wang J, Wu Q, Qian J, Yang C, Bo P (2017). Genistein inhibits the growth and regulates the migration and invasion abilities of melanoma cells via the FAK/paxillin and MAPK pathways. Oncotarget.

[58] Malloy KM, Wang J, Clark LH, Fang Z, Sun W, Yin Y, Kong W, Zhou C, Bae-Jump VL (2018). Novasoy and genistein inhibit endometrial cancer cell proliferation through disruption of the AKT/mTOR and MAPK signaling pathways. Am J Transl Res.

[59] Ge J, Liu Y, Li Q, Guo X, Gu L, Ma ZG, Zhu YP (2013). Resveratrol induces apoptosis and autophagy in T-cell acute lymphoblastic leukemia cells by inhibiting Akt/mTOR and activating p38-MAPK. Biomed Environ Sci.

[60] Salehi B, Mishra AP, Nigam M, Sener B, Kilic M, Sharifi-Rad M, Fokou PVT, Martins N, Sharifi-Rad J (2018). Resveratrol: a double-edged sword in health benefits. Biomedicines.

[61] Zhu J, Yu W, Liu B, Wang Y, Shao J, Wang J, Xia K, Liang C, Fang W, Zhou C (2017). Escin induces caspase-dependent apoptosis and autophagy through the ROS/p38 MAPK signalling pathway in human osteosarcoma cells in vitro and in vivo. Cell Death Dis.

[62] Aldonza MB, Hong JY, Bae SY, Song J, Kim WK, Oh J, Shin Y, Lee SH, Lee SK (2015). Suppression of MAPK signaling and reversal of mTOR-dependent MDR1-associated multidrug resistance by 21alpha-methylmelianodiol in lung cancer cells. PLoS ONE.

[63] Luo W, Liu X, Sun W, Lu JJ, Wang Y, Chen X (2018). Toosendanin, a natural product, inhibited TGF-β1-induced epithelial-mesenchymal transition through ERK/Snail pathway. Phytother Res.

[64] Shao J, Wang C, Li L, Liang H, Dai J, Ling X, Tang H (2018). Luteoloside inhibits proliferation and promotes intrinsic and extrinsic pathway-mediated apoptosis involving MAPK and mTOR signaling pathways in human cervical cancer cells. Int J Mol Sci.

[65] Liang RR, Zhang S, Qi JA, Wang ZD, Li J, Liu PJ, Huang C, Le XF, Yang J, Li ZF (2012). Preferential inhibition of hepatocellular carcinoma by the flavonoid Baicalein through blocking MEK-ERK signaling. Int J Oncol.

[66] Zhang XJ, Jia SS (2016). Fisetin inhibits laryngeal carcinoma through regulation of AKT/NF-kappaB/mTOR and ERK1/2 signaling pathways. Biomed Pharmacother.

[67] Lim W, Park S, Bazer FW, Song G (2017). Naringenin-induced apoptotic cell death in prostate cancer cells is mediated via the PI3K/AKT and MAPK signaling pathways. J Cell Biochem.

[68] Mao J, Yang H, Cui T, Pan P, Kabir N, Chen D, Ma J, Chen X, Chen Y, Yang Y (2018). Combined treatment with sorafenib and silibinin synergistically targets both HCC cells and cancer stem cells by enhanced inhibition of the phosphorylation of STAT3/ERK/AKT. Eur J Pharmacol.

[69] Jain D, Murti Y, Khan WU, Hossain R, Hossain MN, Agrawal KK, Ashraf RA, Islam MT, Janmeda P, Taheri Y (2021). Roles of therapeutic bioactive compounds in hepatocellular carcinoma. Oxid Med Cell Longev.

[70] Oi N, Chen H, Ok Kim M, Lubet RA, Bode AM, Dong Z (2012). Taxifolin suppresses UV-induced skin carcinogenesis by targeting EGFR and PI3K. Cancer Prev Res (Phila).

[71] Kang HM, Park BS, Kang HK, Park HR, Yu SB, Kim IR (2018). Delphinidin induces apoptosis and inhibits epithelial-to-mesenchymal transition via the ERK/p38 MAPK-signaling pathway in human osteosarcoma cell lines. Environ Toxicol.

[72] Lin M, Bi H, Yan Y, Huang W, Zhang G, Zhang G, Tang S, Liu Y, Zhang L, Ma J (2017). Parthenolide suppresses non-small cell lung cancer GLC-82 cells growth via B-Raf/MAPK/Erk pathway. Oncotarget.

[73] Singh VJ, Sharma B, Chawla PA (2021). Recent developments in mitogen activated protein kinase inhibitors as potential anticancer agents. Bioorg Chem.

[74] Pi J, Jiang J, Cai H, Yang F, Jin H, Yang P, Cai J, Chen ZW (2017). GE11 peptide conjugated selenium nanoparticles for EGFR targeted oridonin delivery to achieve enhanced anticancer efficacy by inhibiting EGFR-mediated PI3K/AKT and Ras/Raf/MEK/ERK pathways. Drug Deliv.

[75] Lev-Ari S, Starr A, Vexler A, Karaush V, Loew V, Greif J, Fenig E, Aderka D, Ben-Yosef R (2006). Inhibition of pancreatic and lung adenocarcinoma cell survival by curcumin is associated with increased apoptosis, down-regulation of COX-2 and EGFR and inhibition of Erk1/2 activity. Anticancer Res.

[76] Hao W, Yuan X, Yu L, Gao C, Sun X, Wang D, Zheng Q (2015). Licochalcone A-induced human gastric cancer BGC-823 cells apoptosis by regulating ROS-mediated MAPKs and PI3K/AKT signaling pathways. Sci Rep.

[77] Pan C, Hu Y, Li J, Wang Z, Huang J, Zhang S, Ding L (2014). Estrogen receptor-alpha36 is involved in pterostilbene-induced apoptosis and anti-proliferation in in vitro and in vivo breast cancer. PLoS ONE.

[78] Zhang M, Cai S, Zuo B, Gong W, Tang Z, Zhou D, Weng M, Qin Y, Wang S, Liu J (2017). Arctigenin induced gallbladder cancer senescence through modulating epidermal growth factor receptor pathway. Tumour Biol.

[79] Lee CH, Ying TH, Chiou HL, Hsieh SC, Wen SH, Chou RH, Hsieh YH (2017). Alpha-mangostin induces apoptosis through activation of reactive oxygen species and ASK1/p38 signaling pathway in cervical cancer cells. Oncotarget.

[80] Mi Jeong S, Davaatseren M, Kim W, Sung Kwang P, Kim SH, Haeng Jeon H, Myung Sunny K, Kim YS, Dae Young K (2009). Vitisin A suppresses LPS-induced NO production by inhibiting ERK, p38, and NF-kappaB activation in RAW 2647 cells. Int Immunopharmacol.

[81] Asami Y, Kakeya H, Komi Y, Kojima S, Nishikawa K, Beebe K, Neckers L, Osada H (2008). Azaspirene, a fungal product, inhibits angiogenesis by blocking Raf-1 activation. Cancer Sci.

[82] Polier G, Neumann J, Thuaud F, Ribeiro N, Gelhaus C, Schmidt H, Giaisi M, Köhler R, Müller WW, Proksch P (2012). The natural anticancer compounds rocaglamides inhibit the Raf-MEK-ERK pathway by targeting prohibitin 1 and 2. Chem Biol.

[83] Zhao A, Lee SH, Mojena M, Jenkins R, Patrick DR, Huber HE, Goetz MA, Hensens OD, Zink DL, Vilella D (1999). Resorcylic acid lactones: naturally occurring potent and selective inhibitors of MEK. J Antibiot.

[84] Lee C-J, Lee M-H, Yoo S-M, Choi K-I, Song J-H, Jang J-H, Oh S-R, Ryu H-W, Lee H-S, Surh Y-J (2015). Magnolin inhibits cell migration and invasion by targeting the ERKs/RSK2 signaling pathway. BMC Cancer.

[85] Jeon S, Kim M-M (2019). Tomatidine inhibits cell invasion through the negative modulation of gelatinase and inactivation of p38 and ERK. Chem Biol Interact.

[86] Lim DY, Shin SH, Lee M-H, Malakhova M, Kurinov I, Wu Q, Xu J, Jiang Y, Dong Z, Liu K (2016). A natural small molecule, catechol, induces c-Myc degradation by directly targeting ERK2 in lung cancer. Oncotarget.

[87] Zhi TX, Liu KQ, Cai KY, Zhao YC, Li ZW, Wang X, He XH, Sun XY (2021). Anti-lung cancer activities of 1,2,3-triazole curcumin derivatives via regulation of the MAPK/NF-κB/STAT3 signaling pathways. ChemMedChem.

[88] Kim GD (2017). Kaempferol inhibits angiogenesis by suppressing HIF-1α and VEGFR2 activation via ERK/p38 MAPK and PI3K/Akt/mTOR signaling pathways in endothelial cells. Prev Nutr Food Sci.

[89] Li H, Yoon JH, Won HJ, Ji HS, Yuk HJ, Park KH, Park HY, Jeong TS (2017). Isotrifoliol inhibits pro-inflammatory mediators by suppression of TLR/NF-κB and TLR/MAPK signaling in LPS-induced RAW264.7 cells. Int Immunopharmacol.

[90] Sharifi-Rad J, Quispe C, Imran M, Rauf A, Nadeem M, Gondal TA, Ahmad B, Atif M, Mubarak MS, Sytar O (2021). Genistein: an integrative overview of its mode of action, pharmacological properties, and health benefits. Oxid Med Cell Longev.

[91] Borah N, Gunawardana S, Torres H, McDonnell S, Van Slambrouck S (2017). 5,6,7,3′,4′,5′-Hexamethoxyflavone inhibits growth of triple-negative breast cancer cells via suppression of MAPK and Akt signaling pathways and arresting cell cycle. Int J Oncol.

[92] Sharifi-Rad J, Quispe C, Durazzo A, Lucarini M, Souto EB, Santini A, Imran M, Moussa AY, Mostafa NM, El-Shazly M (2022). Resveratrol’ biotechnological applications: enlightening its antimicrobial and antioxidant properties. J Herb Med.

[93] Wang N, He J, Pan C, Wang J, Ma M, Shi X, Xu Z (2019). Resveratrol activates autophagy via the AKT/mTOR signaling pathway to improve cognitive dysfunction in rats with chronic cerebral hypoperfusion. Front Neurosci.

[94] Zhao W, Lao Y, Liu Y, Niu J, Xiao Z, Arulselvan P, Shen J (2022). Escin induces apoptosis in ovarian cancer cell line by triggering S-phase cell cycle arrest and p38 MAPK/ERK pathway inhibition. J King Saud Univ Sci.

[95] Munakarmi S, Chand L, Shin HB, Hussein UK, Yun B-S, Park HR, Jeong YJ (2020). Anticancer effects of *Poncirus*
*fructus* on hepatocellular carcinoma through regulation of apoptosis, migration, and invasion. Oncol Rep.

[96] Fan W, Fan L, Wang Z, Yang L (2022). Limonoids from the genus Melia (Meliaceae): phytochemistry, synthesis, bioactivities, pharmacokinetics, and toxicology. Front Pharmacol.

[97] Rizzo VL, Levine CB, Wakshlag JJ (2017). The effects of sulforaphane on canine osteosarcoma proliferation and invasion. Vet Comp Oncol.

[98] Sawai Y, Murata H, Horii M, Koto K, Matsui T, Horie N, Tsuji Y, Ashihara E, Maekawa T, Kubo T (2013). Effectiveness of sulforaphane as a radiosensitizer for murine osteosarcoma cells. Oncol Rep.

[99] de Ferreira Oliveria JM, Remédios C, Oliveira H, Pinto P, Pinho F, Pinho S, Costa M, Santos C (2014). Sulforaphane induces DNA damage and mitotic abnormalities in human osteosarcoma MG-63 cells: correlation with cell cycle arrest and apoptosis. Nutr Cancer.

[100] Sharifi-Rad J, Quispe C, Shaheen S, El Haouari M, Azzini E, Butnariu M, Sarac I, Pentea M, Ramírez-Alarcón K, Martorell M (2021). Flavonoids as potential anti-platelet aggregation agents: from biochemistry to health promoting abilities. Crit Rev Food Sci Nutr.

[101] Sharifi-Rad J, Quispe C, Herrera-Bravo J, Martorell M, Sharopov F, Tumer TB, Kurt B, Lankatillake C, Docea AO, Moreira AC (2021). A pharmacological perspective on plant-derived bioactive molecules for epilepsy. Neurochem Res.

[102] Sharifi-Rad J, Bahukhandi A, Dhyani P, Sati P, Capanoglu E, Docea AO, Al-Harrasi A, Dey A, Calina D (2021). Therapeutic potential of neoechinulins and their derivatives: an overview of the molecular mechanisms behind pharmacological activities. Front Nutr.

[103] Salehi B, Prakash Mishra A, Nigam M, Karazhan N, Shukla I, Kiełtyka-Dadasiewicz A, Sawicka B, Głowacka A, Abu-Darwish MS, Hussein Tarawneh A (2021). Ficus plants: state of the art from a phytochemical, pharmacological, and toxicological perspective. Phytother Res.

[104] Sharifi-Rad J, Quispe C, Herrera-Bravo J, Akram M, Abbaass W, Semwal P, Painuli S, Konovalov DA, Alfred MA, Kumar NVA (2021). Phytochemical constituents, biological activities, and health-promoting effects of the *Melissa*
*officinalis*. Oxid Med Cell Longev.

[105] Zhou X, Seto SW, Chang D, Kiat H, Razmovski-Naumovski V, Chan K, Bensoussan A (2016). Synergistic effects of Chinese herbal medicine: a comprehensive review of methodology and current research. Front Pharmacol.

[106] Sharifi-Rad J, Quispe C, Rahavian A, Pereira Carneiro JN, Rocha JE, Alves Borges Leal AL, Bezerra Morais Braga MF, Melo Coutinho HD, Ansari Djafari A, Alarcón-Zapata P (2021). Bioactive compounds as potential agents for sexually transmitted diseases management: a review to explore molecular mechanisms of action. Front Pharmacol.

[107] Salehi B, Rescigno A, Dettori T, Calina D, Docea AO, Singh L, Cebeci F, Özçelik B, Bhia M, Dowlati Beirami A (2020). Avocado-soybean unsaponifiables: a panoply of potentialities to be exploited. Biomolecules.

[108] Hossain R, Quispe C, Herrera-Bravo J, Beltrán JF, Islam MT, Shaheen S, Cruz-Martins N, Martorell M, Kumar M, Sharifi-Rad J (2022). Neurobiological promises of the bitter diterpene lactone andrographolide. Oxid Med Cell Longev.

[109] Guerra B, Issinger O-G (2019). Natural compounds and derivatives as Ser/Thr protein kinase modulators and inhibitors. Pharmaceuticals (Basel).

[110] Lin HH, Robertson KL, Lellupitiyage Don SS, Taylor SR, Farkas ME, Chenoweth DM (2020). Chapter six - chemical modulation of circadian rhythms and assessment of cellular behavior via indirubin and derivatives. Methods in enzymology.

[111] Eisenbrand G, Hippe F, Jakobs S, Muehlbeyer S (2004). Molecular mechanisms of indirubin and its derivatives: novel anticancer molecules with their origin in traditional Chinese phytomedicine. J Cancer Res Clin Oncol.

[112] Abdallah QA, Fortwendel JR (2015). Exploration of *Aspergillus*
*fumigatus* Ras pathways for novel antifungal drug targets. Front Microbiol.

[113] Song J-H, Lee C-J, An H-J, Yoo S-M, Kang HC, Lee JY, Kim KD, Kim DJ, Lee HS, Cho Y-Y (2019). Magnolin targeting of ERK1/2 inhibits cell proliferation and colony growth by induction of cellular senescence in ovarian cancer cells. Mol Carcinog.

[114] Calina D, Buga AM, Mitroi M, Buha A, Caruntu C, Scheau C, Bouyahya A, El Omari N, El Menyiy N, Docea AO (2020). The treatment of cognitive, behavioural and motor impairments from brain injury and neurodegenerative diseases through cannabinoid system modulation-evidence from in vivo studies. J Clin Med.

[115] Sharifi-Rad J, Quispe C, Herrera-Bravo J, Martorell M, Sharopov F, Tumer TB, Kurt B, Lankatillake C, Docea AO, Moreira AC (2021). A pharmacological perspective on plant-derived bioactive molecules for epilepsy. Neurochem Res.

[116] Sharifi-Rad J, Quispe C, Kumar M, Akram M, Amin M, Iqbal M, Koirala N, Sytar O, Kregiel D, Nicola S (2022). Hyssopus essential oil: an update of its phytochemistry, biological activities, and safety profile. Oxid Med Cell Longev.

[117] Islam MT, Quispe C, El-Kersh DM, Shill MC, Bhardwaj K, Bhardwaj P, Sharifi-Rad J, Martorell M, Hossain R, Al-Harrasi A (2021). A literature-based update on *Benincasa*
*hispida* (Thunb.) Cogn.: traditional uses, nutraceutical, and phytopharmacological profiles. Oxid Med Cell Longev.

[118] Hossain R, Quispe C, Herrera-Bravo J, Islam MS, Sarkar C, Islam MT, Martorell M, Cruz-Martins N, Al-Harrasi A, Al-Rawahi A (2021). *Lasia*
*spinosa* chemical composition and therapeutic potential: a literature-based review. Oxid Med Cell Longev.

[119] Salehi B, Quispe C, Chamkhi I, El Omari N, Balahbib A, Sharifi-Rad J, Bouyahya A, Akram M, Iqbal M, Docea AO (2021). Pharmacological properties of chalcones: a review of preclinical including molecular mechanisms and clinical evidence. Front Pharmacol.

[120] Ji HF, Li XJ, Zhang HY (2009). Natural products and drug discovery. Can thousands of years of ancient medical knowledge lead us to new and powerful drug combinations in the fight against cancer and dementia?. EMBO Rep.

[121] Safarzadeh E, Sandoghchian Shotorbani S, Baradaran B (2014). Herbal medicine as inducers of apoptosis in cancer treatment. Adv Pharm Bull.

[122] Sharifi-Rad J, Kamiloglu S, Yeskaliyeva B, Beyatli A, Alfred MA, Salehi B, Calina D, Docea AO, Imran M, Kumar NVA (2020). Pharmacological activities of psoralidin: a comprehensive review of the molecular mechanisms of action. Front Pharmacol.

[123] Baba Y, Kato Y (2017). Deguelin, a novel anti-tumorigenic agent in human esophageal squamous cell carcinoma. EBioMedicine.

[124] Ma C, Peng Y, Li H, Chen W (2021). Organ-on-a-chip: a new paradigm for drug development. Trends Pharmacol Sci.

[125] Roskoski R (2021). Properties of FDA-approved small molecule protein kinase inhibitors: a 2021 update. Pharmacol Res.

[126] Shepherd C, Puzanov I, Sosman JA (2010). B-RAF inhibitors: an evolving role in the therapy of malignant melanoma. Curr Oncol Rep.

[127] Singh OV, Gabani P (2011). Extremophiles: radiation resistance microbial reserves and therapeutic implications. J Appl Microbiol.

[128] Salehi B, Sharifi-Rad J, Capanoglu E, Adrar N, Catalkaya G, Shaheen S, Jaffer M, Giri L, Suyal R, Jugran AK (2019). Cucurbita plants: from farm to industry. Appl Sci-Basel.

[129] Popović-Djordjević J, Quispe C, Giordo R, Kostić A, Katanić Stanković JS, Tsouh Fokou PV, Carbone K, Martorell M, Kumar M, Pintus G (2022). Natural products and synthetic analogues against HIV: a perspective to develop new potential anti-HIV drugs. Eur J Med Chem.

[130] Quispe C, Herrera-Bravo J, Javed Z, Khan K, Raza S, Gulsunoglu-Konuskan Z, Daştan SD, Sytar O, Martorell M, Sharifi-Rad J (2022). Therapeutic applications of curcumin in diabetes: a review and perspective. Biomed Res Int.

[131] Tsoukalas D, Fragkiadaki P, Docea AO, Alegakis AK, Sarandi E, Vakonaki E, Salataj E, Kouvidi E, Nikitovic D, Kovatsi L (2019). Association of nutraceutical supplements with longer telomere length. Int J Mol Med.

[132] Tsoukalas D, Zlatian O, Mitroi M, Renieri E, Tsatsakis A, Izotov BN, Burada F, Sosoi S, Burada E, Buga AM (2021). A novel nutraceutical formulation can improve motor activity and decrease the stress level in a murine model of middle-age animals. J Clin Med.

[133] Salehi B, Sestito S, Rapposelli S, Peron G, Calina D, Sharifi-Rad M, Sharopov F, Martins N, Sharifi-Rad J (2019). Epibatidine: a promising natural alkaloid in health. Biomolecules.

